# A Rare Association of Emphysematous Gastritis With Gastric Adenocarcinoma: A Case Report and Diagnostic Pitfalls

**DOI:** 10.7759/cureus.107492

**Published:** 2026-04-21

**Authors:** Garrett Teskey, Melanie De Shadarevian, Wesley Chow, Steven J Gauerke, Steven Wong

**Affiliations:** 1 Internal Medicine, Scripps Mercy Hospital, San Diego, USA; 2 Pathology, Scripps Mercy Hospital, San Diego, USA; 3 Critical Care Medicine, Scripps Mercy Hospital, San Diego, USA

**Keywords:** diagnostic challenge, emphysematous gastritis, gastric adenocarcinoma, gastroenterology, malignancy

## Abstract

Emphysematous gastritis is a rare and life-threatening condition characterized by the presence of gas within the gastric wall, typically caused by gas-forming microorganisms. It is thought to arise from the disruption of mucosal integrity in the setting of underlying predisposing factors. Given its high mortality rate, early recognition and prompt management are critical to improving outcomes. We present a rare case of emphysematous gastritis secondary to advanced gastric adenocarcinoma. A limited number of cases describing an association between emphysematous gastritis and malignancy have been reported in the literature, with only a small subset linked to gastric adenocarcinoma. This case underscores the importance of maintaining a high index of suspicion for atypical and malignant etiologies, particularly in patients with nonspecific presentations, as delayed diagnosis may result in rapid clinical deterioration and poor outcomes.

## Introduction

Emphysematous gastritis is a rare and often fatal inflammatory condition of the gastric wall characterized by the presence of gas-producing microorganisms. Common causative organisms include *Streptococcus* species, *Escherichia coli*, *Enterobacter* species, *Clostridium* species, *Staphylococcus aureus*, *Klebsiella pneumoniae*, *Pseudomonas aeruginosa*, and *Candida* species [1,2]. Patients typically present with acute abdominal pain, nausea, and vomiting, and may rapidly progress to septic shock. Reported mortality rates approach 60%, underscoring the severity of this condition [3-5].

The pathogenesis is thought to involve disruption of the gastric mucosal barrier, allowing invasion of gas-forming organisms into the gastric wall. This may occur through direct mucosal injury, ischemia, or hematogenous spread. Several predisposing factors have been identified, including corrosive ingestion, recent surgery, bowel obstruction, gastric distension, vomiting, steroid use, immunosuppression, chemotherapy, alcohol use, and nonsteroidal anti-inflammatory drug exposure [1-3].

Computed tomography (CT) is the imaging modality of choice and is critical for early diagnosis, as it can reliably detect intramural gas within the stomach wall. Despite advances in imaging and management, diagnosis remains challenging due to the nonspecific clinical presentation and the rarity of the condition.

Given these challenges, recognition of atypical presentations and uncommon underlying etiologies is essential. Here, we present a rare case of emphysematous gastritis associated with advanced gastric adenocarcinoma, highlighting the diagnostic complexity and rapid progression of this life-threatening condition.

This article was previously presented as a meeting abstract at the 2025 ACG Annual Scientific Meeting on October 28, 2025 [6].

## Case presentation

A 60-year-old male presented to the emergency department with one week of right upper quadrant abdominal pain. He reported several months of severe postprandial cramping pain, worsened by fatty or spicy foods. He also noted an unintentional 10-15-pound weight loss approximately one month before presentation. Additional symptoms included two episodes of nonbilious, nonbloody emesis without diarrhea, constipation, or changes in bowel or bladder habits.

Two years prior, esophagogastroduodenoscopy revealed a short segment of Barrett’s esophagus, and a same-day colonoscopy demonstrated benign polyps, which were removed with a recommendation for seven-year follow-up. The patient also underwent routine abdominal CT imaging for a complex renal history, including hypospadias, congenital left ureteral stenosis, recurrent gross hematuria requiring cauterization, and left hydroureteronephrosis. A CT scan performed six months before admission showed no gastrointestinal abnormalities. The day before admission, an outpatient ultrasound demonstrated a 10 mm dilation of the common bile duct.

Initial laboratory studies were notable for elevated alkaline phosphatase, microcytic anemia, and new-onset transaminitis. The calculated R factor was 0.6, consistent with a cholestatic pattern of injury [7]. Repeat right upper quadrant ultrasound demonstrated nonspecific gallbladder wall thickening and dilation of the common bile duct to 13 mm, without cholelithiasis. Subsequent magnetic resonance cholangiopancreatography (MRCP) showed mild dilation of the biliary and pancreatic ducts without evidence of obstruction. General surgery recommended against cholecystectomy and advised hepatobiliary iminodiacetic acid (HIDA) scan evaluation, which was unremarkable. Gastroenterology was subsequently consulted for further evaluation.

**Figure 1:**
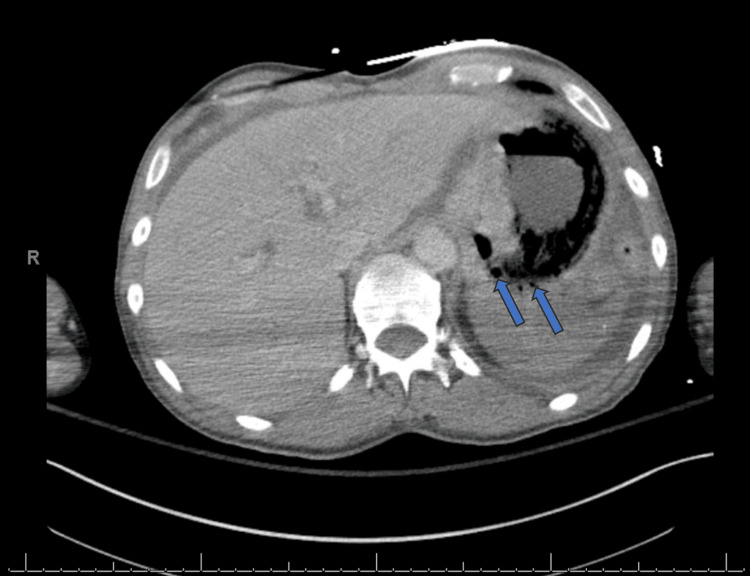
Computed tomography of the abdomen and pelvis showing emphysematous gastritis (arrows).

Before additional workup could be completed, the patient acutely decompensated. A rapid response was initiated for unresponsiveness and hypotension, with a blood pressure of 75/59 mmHg. He was intubated, started on vasopressor support, and transferred to the intensive care unit. Following stabilization, gastroenterology recommended an urgent CT of the abdomen and pelvis with intravenous contrast to evaluate for an alternative etiology. This imaging revealed emphysematous gastritis with a 7.4 × 14.0 cm gas-containing, rim-enhancing perigastric fluid collection (Figure 1). Additional findings included pneumobilia, pneumoperitoneum, and global hypoenhancement of the spleen.

**Figure 2:**
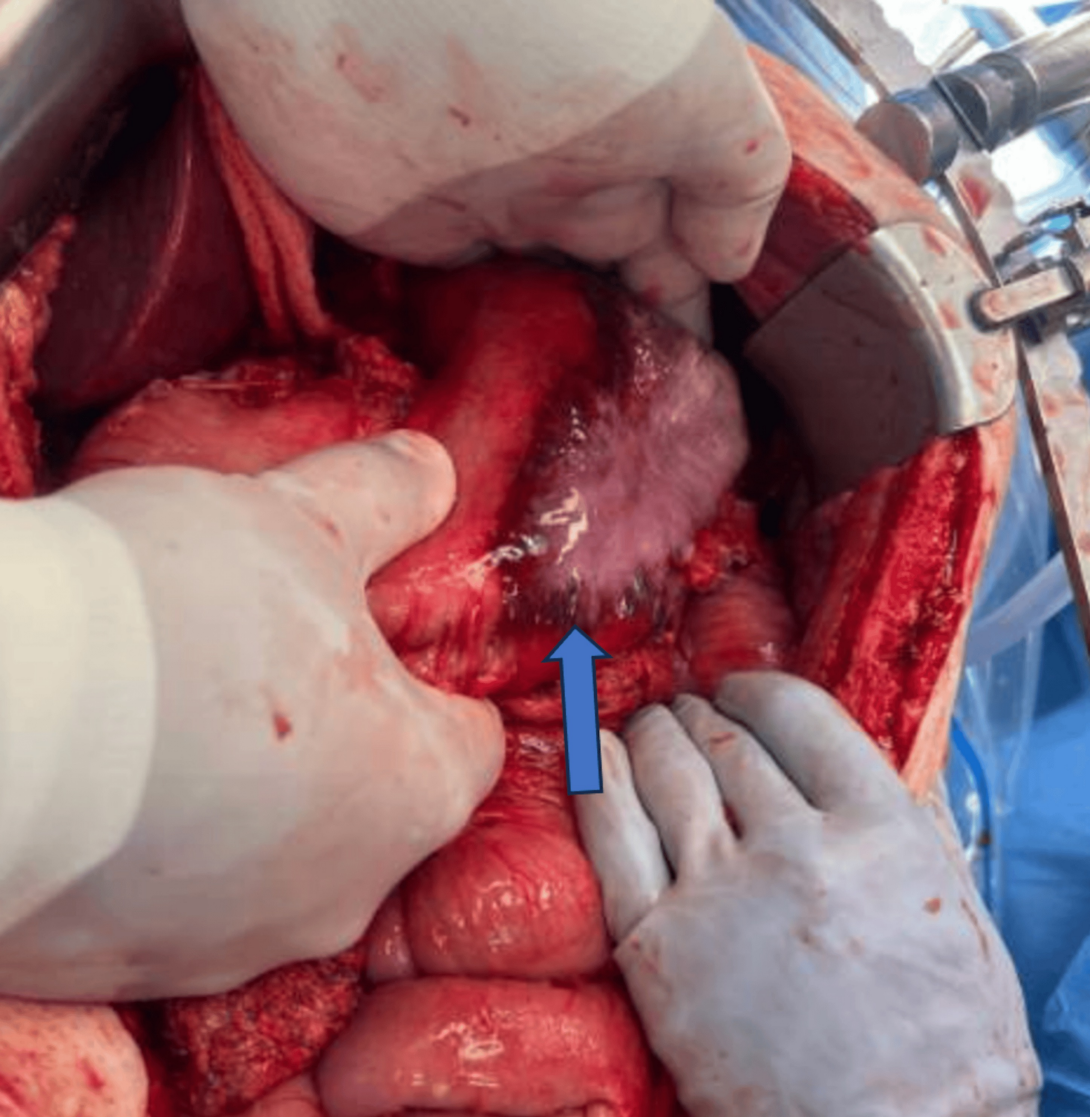
Gross examination of the stomach revealing diffuse inflammation and thickening of the stomach wall, along with a large area of gastric ischemia and necrosis, and no visible perforation observed.

Given these findings and the patient’s clinical instability, the decision was made to proceed with emergent exploratory laparotomy. Intraoperatively, purulent fluid was identified in the left upper abdomen, along with diffuse gastric wall inflammation and thickening. A large area of gastric ischemia and necrosis was present without visible perforation. Frozen section biopsy demonstrated spindle cells, prompting intraoperative surgical oncology consultation. Due to the extent of necrosis, a subtotal gastrectomy was performed (Figure 2). Temporary abdominal closure was achieved using an ABThera negative-pressure wound therapy system (vacuum-assisted closure).

**Figure 3:**
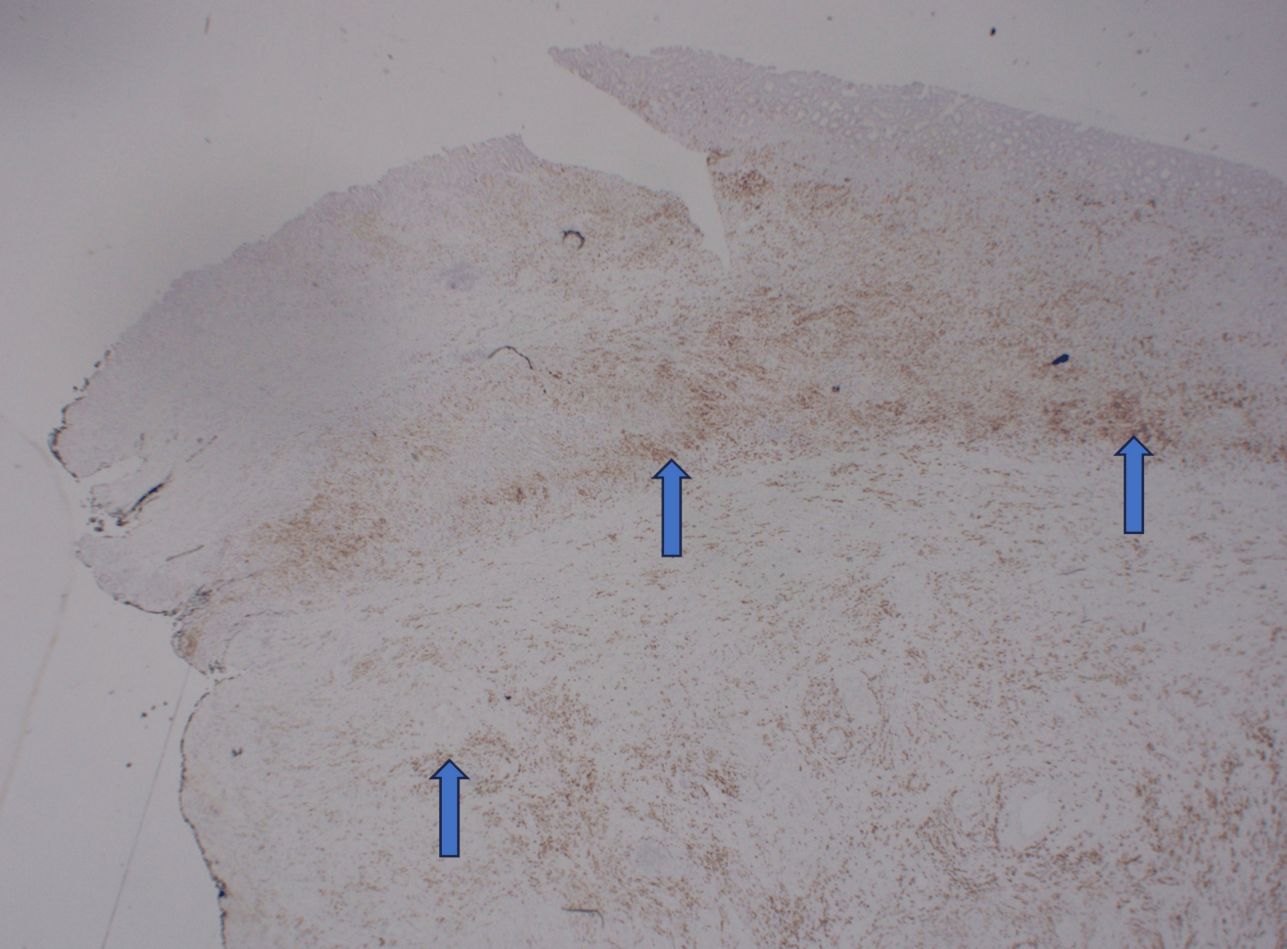
Immunohistochemistry of the gastric tissue biopsy demonstrating positive villin staining, which is highly sensitive for gastrointestinal adenocarcinomas.

**Figure 4:**
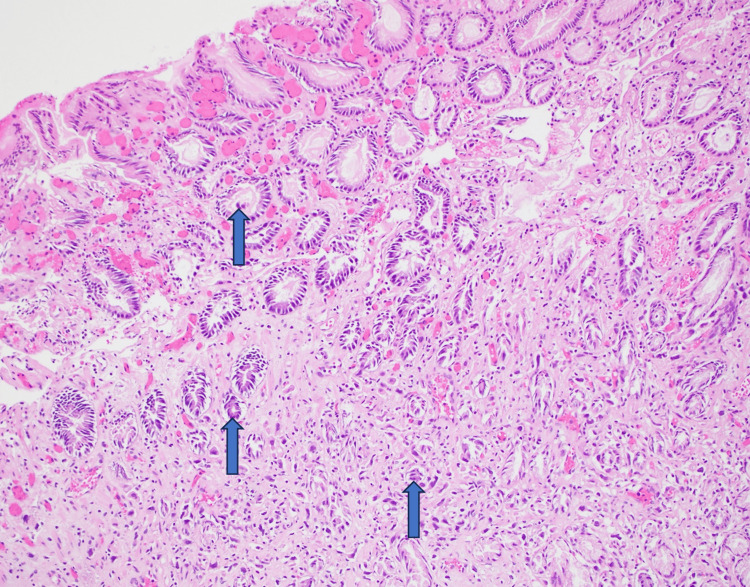
Hematoxylin and eosin staining of the gastric antrum biopsy demonstrating abnormal cell morphology, including enlarged nuclei, increased cellular density, and irregular tissue architecture.

**Figure 5:**
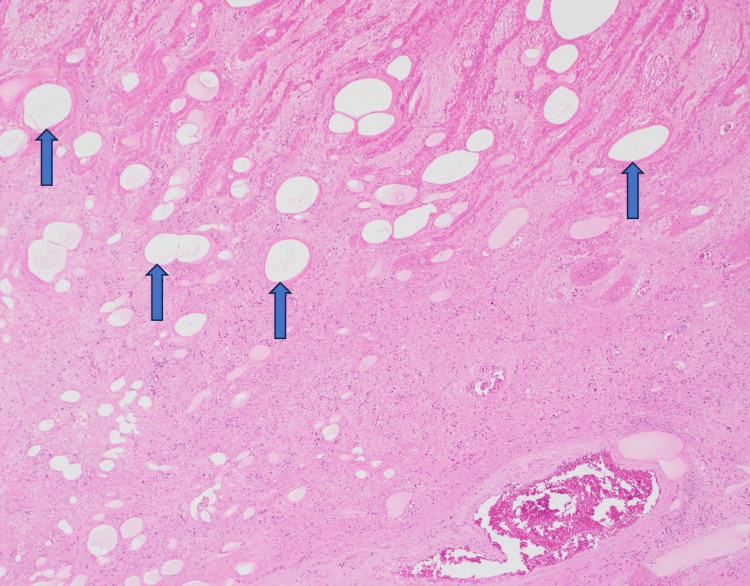
Low-power magnification of a gastric biopsy demonstrating abundant empty spaces without cellular lining within necrotic tissue, consistent with emphysematous gastritis.

Final pathology demonstrated poorly differentiated gastric adenocarcinoma with signet-ring features, staining positive for villin, CK7, CK20, CDH17, and CDX2 (Figures 3, 4). Histopathologic evaluation further revealed abundant empty spaces lacking cellular lining within necrotic tissue, consistent with emphysematous gastritis; however, no identifiable organisms were seen (Figure 5). Intraoperatively, the patient experienced hemodynamic instability, requiring splenectomy to address ongoing bleeding in the left upper quadrant and a partial colectomy of the transverse colon due to ischemia. Postoperatively, neurologic examination revealed fixed and dilated pupils with no response to noxious stimuli. CT of the head demonstrated severe cerebral edema with tonsillar herniation, consistent with irreversible brain injury. After discussions with family and care teams, the patient was transitioned to comfort measures and subsequently expired.

## Discussion

Emphysematous gastritis is a rare and highly lethal condition characterized by the presence of gas within the gastric wall [1]. Clinical presentation is often nonspecific, including abdominal pain, nausea, and vomiting, with severity ranging from mild discomfort to fulminant sepsis and shock [8-10]. This variability, combined with the rarity of the condition, may contribute to delays in diagnosis. In this case, advanced gastric adenocarcinoma likely contributed to mucosal compromise and local ischemia, creating a permissive environment for gas formation within the gastric wall. This highlights an important but uncommon pathophysiologic link between malignancy and emphysematous gastritis.

The patient’s initial presentation and workup suggested a hepatobiliary etiology, which guided early management. However, nondiagnostic abdominal ultrasound, MRCP, and HIDA scan findings delayed consideration of alternative diagnoses. Notably, CT imaging, the preferred modality for evaluation, was not performed early on, further contributing to diagnostic delay. This case underscores the importance of early cross-sectional imaging in patients with persistent or unexplained abdominal pain, particularly when initial studies are inconclusive.

Management of emphysematous gastritis typically includes early broad-spectrum antibiotics, hemodynamic support, and close monitoring. While some cases may resolve with medical management alone when diagnosed early, advanced disease is often associated with gastric necrosis and poor outcomes [9-12]. Surgical intervention is generally reserved for complications such as perforation, necrosis, or clinical deterioration [13]. In this patient, extensive gastric ischemia and necrosis necessitated emergent surgical intervention; however, despite aggressive management, the disease proved fatal.

This case reinforces several key clinical implications. First, emphysematous gastritis should remain in the differential diagnosis for patients with severe or unexplained abdominal pain. Second, underlying malignancy should be considered a potential precipitating factor, particularly in patients with unexplained weight loss or atypical presentations. Finally, early recognition and prompt imaging are critical, as delays in diagnosis may lead to rapid clinical deterioration and high mortality.

## Conclusions

This case highlights a rare and aggressive presentation of emphysematous gastritis associated with advanced gastric adenocarcinoma. Although only a limited number of cases have been reported linking emphysematous gastritis to malignancy, this association carries significant clinical implications. Given the condition’s high mortality and rapid progression, early recognition and prompt intervention are critical. The nonspecific presentation may obscure the diagnosis, underscoring the importance of early cross-sectional imaging and timely reassessment when initial evaluations are nondiagnostic. In this patient, an initial focus on a biliary etiology delayed definitive diagnosis, contributing to rapid clinical deterioration and death. This case emphasizes the need for a high index of suspicion and a broad differential diagnosis to improve outcomes in this life-threatening condition.

## References

[1] Matsushima K, Won EJ, Tangel MR, Enomoto LM, Avella DM, Soybel DI (2015). Emphysematous gastritis and gastric emphysema: similar radiographic findings, distinct clinical entities. World J Surg.

[2] Yalamanchili M, Cady W (2003). Emphysematous gastritis in a hemodialysis patient. South Med J.

[3] Ashfaq A, Chapital AB (2015). Emphysematous gastritis in a patient with coxsackie B3 myocarditis and cardiogenic shock requiring veno-arterial extra-corporeal membrane oxygenation. Int J Surg Case Rep.

[4] Caruana V, Swayne LC, Salaki JS (1990). Indium-111 WBC detection of emphysematous gastritis in pancreatitis. J Nucl Med.

[5] Watson A, Bul V, Staudacher J, Carroll R, Yazici C (2017). The predictors of mortality and secular changes in management strategies in emphysematous gastritis. Clin Res Hepatol Gastroenterol.

[6] Teskey G, Chow W, Lopez A, Wong S, Gauerke S, De Shadarevian M (2025). A rare case of emphysematous gastritis precipitated by advanced gastric adenocarcinoma. Am J Gastroenterol.

[7] Chalasani NP, Maddur H, Russo MW, Wong RJ, Reddy KR (2021). ACG Clinical Guideline: diagnosis and management of idiosyncratic drug-induced liver injury. Am J Gastroenterol.

[8] Qasim A, Penikelapate S, Sosa F, Jyala A, Ghazanfar H, Patel H, Dev A (2023). Emphysematous gastritis: a case series on a rare but critical gastrointestinal condition. Cureus.

[9] Nasser H, Ivanics T, Leonard-Murali S, Shakaroun D, Woodward A (2019). Emphysematous gastritis: a case series of three patients managed conservatively. Int J Surg Case Rep.

[10] Ghasemi F, Kirkpatrick I, Sharma A (2023). Medical management of emphysematous gastritis. Ann Intern Med Clin Cases.

[11] Zamora Elson M, Labarta Monzón L, Escos Orta J, Cambra Fierro P, Vernal Monteverde V, Seron Arbeloa C (2016). [Emphysematous gastritis: effectiveness of early antibiotic therapy]. Gastroenterol Hepatol.

[12] Vanegas-Duarte E, Duque-Montaño AM (2021). Emphysematous gastritis in association with gastric adenocarcinoma. A case report. Rev Colomb Gastroenterol.

[13] Riaz S, Kudaravalli P, Saleem SA, Sapkota B (2020). Emphysematous gastritis: a real indication for emergent surgical intervention?. Cureus.

